# 

**Published:** 1921-04-02

**Authors:** 


### Gifts and Bequests.

Under the will of Sir Edwin Galsworthy, for nearly twenty years Chairman of the Metropolitan Asylums Board, the London Fever Hospital and the Temperance Hospital will receive £100 each, and the Victoria Hospital for Children, Chelsea, and the Royal Free Hospital £50 each.

Mrs. Ellen Barnes, of Weston Rhyn, Salop, left £3,000 in trust for the benefit of charities or institutions of an educational, religious, or philanthropic nature in the neighbourhood of Quinta or adjoining parishes, as the trustees in their discretion may select.

### Passing Events and Topics.

### Where Voluntary Centres Flourish.

According to a report of the Southampton County Council's Health Committee, the work in connection with maternity and child welfare in Hampshire has increased very rapidly, and there are no fewer than forty-five centres in existence at present. In the majority of cases these centres are not only run on voluntary lines, but are managed at a minimum of expense to the County Council. When the first centres were established the question of financing them was one of no little difficulty, the expenditure often being made up by a considerable number of trifling items which could hardly have been dealt with by way of accounts submitted to the quarterly meeting of the County Council. Moreover, the close supervision of petty details which such a procedure would have entailed would have been irksome to the local workers, and would have been incompatible with the successful working of voluntary institutions. It is, generally speaking, not possible to carry on work on voluntary lines without giving the volunteers a certain amount of authority and liberty. It has been the practice for the last three years to make small grants to the respective centres, and the total authorised under this heading is only £300 for forty-five centres. Regarded from a financial point of view, the method is a very successful one.

### Anti-Venereal Campaign in Shanghai.

The Commission appointed by the National Council for Combating Venereal Diseases and sent out to the Far East under the ægis of the Colonial Office, has just completed a strenuous campaign in Shanghai, which the Commission reached early in December. The Shanghai Municipal Council had appointed an Advisory Committee to make arrangements for the stay of the Commission, and the campaign opened with a representative Conference convened by the Council. Publicity was secured through the medium of the press and by the issue of handbills through the principal firms and voluntary organisations. It is believed that as a result of the campaign much of the programme recommended by the Commissioners will be carried out, and that in any case the appointment of a V.D. officer and the provision of free treatment for seafarers will be considered in the immediate future.

### "What's-a-Name" Competition.

The organisers of the Theatrical Garden-Party in aid of the Actors' Orphanage have decided to seek a new way for ensuring the success of this year's Party. Appreciating the publicity value of a striking catch-phrase, they ask the public to provide them with one by taking part in a grand "What's-a-Name" competition. In other words, for the purpose of getting help to sell their Garden Party tickets, they are offering £1,000 in prizes, with a first prize of £500, for the best suggestion. It must consist of not more than eight words. In order to take part in the competition a one-shilling entrance coupon (to be had at all the principal theatres and cinemas, and from most of the popular artistes), or a shilling Postal Order must be enclosed with the phrase, and sent to the offices at 3 Middle Temple Lane, E.C. 4. A phrase (not for competition) sent in by Miss Marie Lloyd is "Happiness Fair for Happiness House."

### Municipal Dentistry.

Bermondsey Borough Council have established a municipal dentistry, which promises to be of exceedingly good service to the community, but at present it is limited under the Act to children under five years of age and nursing and expectant mothers. Under Section 77 of the Public Health (London) Act, 1891, however, there seems, according to Dr. King Brown, a possibility of extending the treatment to mothers who are attending the Centres in connection with their children, but who are not necessarily breast-feeding or expectant. The Council has therefore applied to the Ministry of Health to extend the scope of the new department.

### Six Months' Tuberculosis Work.

Dr. Priestley, the Medical Officer of Health for Lambeth, has prepared a report of the work during the past six months at the two tuberculosis dispensaries. This shows 972 first and 5,970 other attendances. Visits paid to patients' homes numbered 2,251, and 884 cases were referred to affiliated hospitals. Of the 972 new cases 278 proved to be suffering from pulmonary tuberculosis and forty-one from non-pulmonary tuberculosis. Those found to be non-tuberculous, from physical signs, were 411, whilst 242 are classified as doubtful.

**RATES OF SUBSCRIPTION** (post free, payable in advance).
Three months, 3/3; six months, 6/6; twelve months, 13/-. (Should the cost of printing and paper as well as increased postage, necessitate alteration of these rates, the period paid for will be reduced proportionately on unexpired subscriptions.) THE HOSPITAL "Index" to Volume LXVIII., April to September 1920, is now ready, price 1/1 per copy, post free. Address The Manager, THE HOSPITAL, "The Hospital" Building, 28/29 Southampton Street, Strand, London, W.C. 2.

